# Simulating large-scale biotech bubble columns: challenges and insights from the parcel approach

**DOI:** 10.1007/s00449-026-03397-5

**Published:** 2026-08-08

**Authors:** Carolin Link, Lars Puiman, Ralf Takors

**Affiliations:** 1 Institute of Biochemical Engineering, University of Stuttgart, Allmandring 31, 70569 Stuttgart, Germany; 2 Australian Institute for Bioengineering and Nanotechnology, The University of Queensland, Corner Cooper Rd & College Rd, St. Lucia, Brisbane, QLD 4072 Australia; 3 ARC Centre of Excellence in Synthetic Biology, The University of Queensland, St. Lucia, Brisbane, QLD 4072 Australia

**Keywords:** Lattice Boltzmann, Euler-Lagrange, Parcel approach, CFD

## Abstract

The growing industrial demand for economically attractive large-scale bioprocesses has increased the need for reliable and computationally feasible modelling approaches. Gas fermentation plays a particular role as the targeted commodities ask for large-scale solutions using bubble-column type bioreactors that allow them to benefit from the economy-of-scale. To reduce development efforts, large-scale designs increasingly build on computational fluid dynamics (CFD) simulations. State-of-the-art approaches employ finite-volume Reynolds-averaged Navier-Stokes (FV-RANS) methods that require enormous computational efforts. In contrast, Euler-Lagrange (E-L) approaches offer detailed local resolution as a prerequisite for optimum bioreactor design. By tracking individual bubbles, they predict bubble population dynamics while computations are parallelized. However, to cope with realistic bubble numbers, large-scale simulations typically make use of the so-called ‘parcel size’ approach, i.e. multiple bubbles are represented by a single computational particle. This study applies Lattice Boltzmann large eddy simulations (LB-LES) to illustrate the observed consequences: Swarm-like bubble behavior is found that results in inhomogeneous bubble distributions. As a consequence, the volumetric mass transfer coefficient *k*_*L*_*a*, a key criterion for design, may be biased. Comparing results with state-of-the-art FV-RANS simulations revealed similarities of transient flow structures and averaged velocity values. However, local deviations are found that mirror handling differences of turbulence representation and the impact of the parcel approach. The findings demonstrate the potential and current limitations of parcel-based modelling for large-scale bioreactors.

**Supplementary Information:**

The online version contains supplementary material available at https://doi.org/10.1007/s00449-026-03397-5.

## Introduction

In many industrial sectors, including pharmaceuticals, beverages, and food ingredients, interest in bioprocesses has grown steadily over the years [1], driving the development of industrial-scale facilities. In particular, low-margin products benefit from the economy-of-scale principle to become profitable [2, 3]. Since experimental investigations and scale-up are time- and resource-intensive, modeling approaches provide a valuable alternative by accelerating process design, enabling insights into large-scale systems, and decreasing the time-to-market [4].

Computational Fluid Dynamics (CFD) became an important and efficient tool for the design and optimization of industrial (bio-)reactors, giving information on the complex hydrodynamics, mixing, gas dispersion and substrate consumption in bioprocesses, revealing gradients that affect the process outcome [2, 5].

A wide range of CFD-based reactor models have been published (see Table 1) but simulations are computationally expensive and experimental data for model validation of industrial-scale tanks is usually missing [2, 6].

**Table 1:** Representative list of industrial-scale reactor models published in literature with the applied modelling method and simulation framework

Reactor	Simulation framework	Reference
600 m^3^ BCR	Data-based CM	[7]
600 m^3^ BCR/ALR	Euler-Lagrange, LB-LES (M-Star), parcel size 500	[2]
565 m^3^ EL-ALR	Euler-Euler, RANS (Ansys)	[8]
125 m^3^ BCR	Euler-Euler, RANS (Ansys)	[9]
100 m^3^ STR	Euler-Euler, RANS and CM	[6]
Up to 90 m^3^ STR, BCR	Euler-Euler, RANS	[10]
up to 75 m^3^ STR	Euler-Euler, RANS (Ansys), PBM	[11]
54 m^3^ STR	Euler-Euler, RANS (Ansys)	[12]
up to 40 m^3^	Euler-Lagrange, LB-LES	[13]
22 m^3^ STR	Euler-Euler, RANS (Ansys)	[14, 15]
4.1 m^3^ STR	Euler-Euler, RANS (Ansys)	[16]
4.1 m^3^ BCR	Euler-Euler, RANS (Ansys)	[17]
up to 1.5 m^3^ STR	Euler-Euler, RANS (Ansys) and CM	[18]

*RANS* Reynolds-averaged Navier-Stokes, *CM* compartment model, *LES* Large eddy simulation, *PBM* Population balance model, *STR* Stirred tank reactor, *BCR* Bubble column reactor

While numerous studies have focused on the simulation of stirred tank reactors (STRs) in industrial-scale [11, 12, 14, 15], more recent work extended the modeling efforts to industrial scale bubble column bioreactors (BCRs) [2, 7–9]. BCRs offer distinct advantages for industrial applications, including enhanced mixing, higher gas holdups [10], and lower (aeration) costs [19] compared to stirred tanks. This makes BCR-type bioreactors particularly attractive for gas fermentation approaches as already indicated in Table 1. Given that often low margin commodities are produced by gas fermentation, economy-of-scale starts benefitting for bioreactor volumes exceeding 500 m³. For comparison, chemical reactors larger than 1 m^3^ may already be considered as industrial-scale for most applications [16]. In chemical production, very large size reactors comprise 100 to 200 m^3^ [20] mirroring the higher volumetric chemical reaction rates compared to biochemical applications. In contrast, largest biotech BCRs of 10,000 m³ are found for wastewater treatment [20].

With the rise of gas fermentation technologies, BCRs have become a central focus of research. Although various modeling approaches – such as Euler-Euler (E-E) Reynolds-averaged Navier-Stokes (RANS) [8] and Euler-Lagrange (E-L) Large eddy simulations (LES) [2] – have been applied to industrial-scale BCRs, their relative accuracy, critical assumptions, and suitability for pneumatically agitated systems remain unclear. Most studies rely on Finite volume (FV)-RANS models [8, 9, 12, 15], though Lattice Boltzmann (LB)-LES simulations have been increasingly employed in recent years [2, 21]. Furthermore, much progress is achieved for thoroughly applying large-scale compartmentation approaches [22, 23]. To better understand the influence of the modeling approaches, key definitions are introduced in Sect. 2.

LB-LES has been found to lead to significant increase in computational speed for stirred [21] and non-stirred reactors [2]. However, the simulation of BCRs with high gas holdups holds its own challenges. Enhanced parallelization and improved computational resources accelerated CFD simulations based on the LBM in recent years. Therewith, the predictability of the highly transient and dynamic nature of bioreactors by advanced turbulence modeling via LES increased. However, certain limitations remain. One important aspect is the Lagrangian perspective on bubbles, meaning that each bubble is individually tracked. While the approach enables detailed investigation of bubble dynamics [2], it is not possible to track all bubbles, if the number of bubbles exceeds the memory that is available on in-house workstations, or high-end servers. As a workaround, the parcel approach (also called coarse-graining approach [24] is often applied representing a pre-defined number of bubbles by a single parcel. The diameter of the parcel is the same as the diameter of the represented bubbles. The influence of this parcel on the liquid is scaled with respect to the number of lumped bubbles. In other disciplines, the impact of parcel sizes has already been investigated. Research studies on sprays already pointed out that the parcel size (or ratio) has to be selected carefully in order to avoid underestimations [25]. Ren et al. already critically evaluated scaling rules based on coarse-grained CFD-DEM simulations for fluidized beds with stiff particles [24]. However, the transfer of findings to bubble dynamics is hampered due to the different nature of the particles. On contrary, Thomas et al. showed that the parcel size has no significant impact on the flow field in bioreactors as long as the number of tracked parcels is still statistically relevant [26]. However, this study focused on STRs where mechanical power input by stirring dominates fluid flows. In BCRs, the liquid is mainly governed by the motion of the bubbles. Exactly this feature might influence the simulation results more drastically than in STRs.

Consequently, our study sets out to investigate the putative impact of parcel size on a set of characteristic performance criteria. E-E RANS simulations of an industrial-scale external loop airlift reactor (EL-ALR [8]), serve as reference for comparison with E-L LB-LES (i.e., regarding upward liquid velocity, gas holdup and energy dissipation rate). Considering that LB-LES basically enables the prediction of locally resolved turbulence, the study thus investigates the potential drawback of the parcel approach for industrial scale simulations. In addition to quantifying the effect of parcel size, the study aims to systematically evaluate the respective advantages and drawbacks of both modelling approaches with respect to predicting key hydrodynamic quantities. Insights are intended to enable bioprocess engineers choosing optimal model settings for predicting gas-liquid mass transfer. This is a prerequisite for the proper design of industrial scale gas fermentation.

## Theory and definitions

**Fig. 1:**
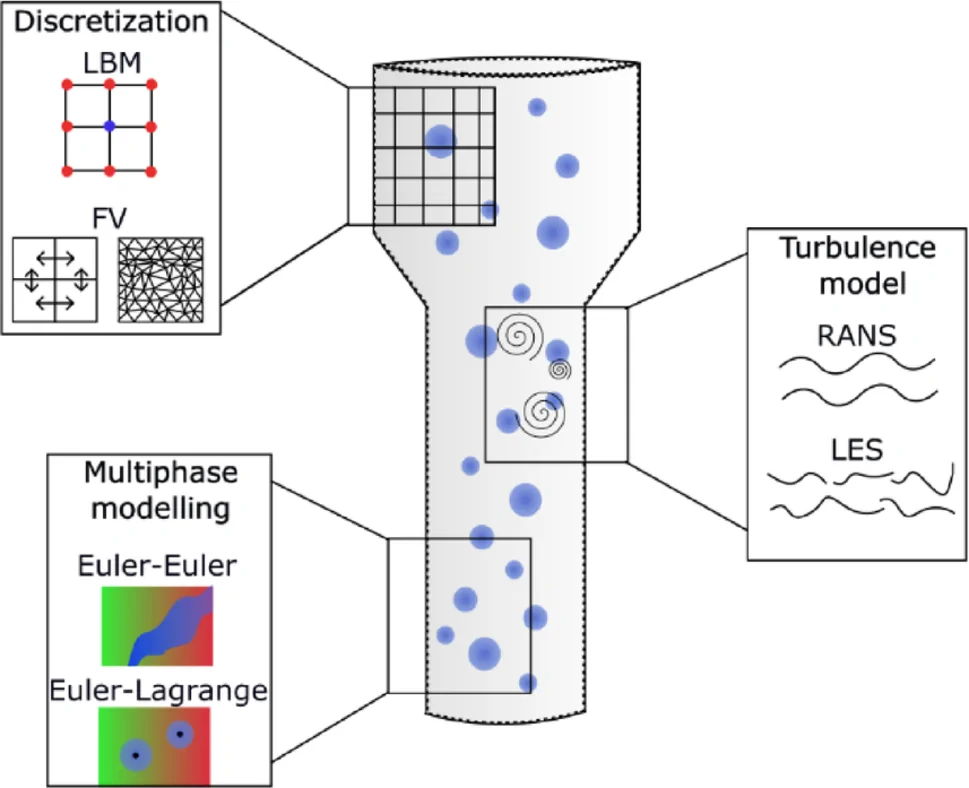
Considerations and model choices for the computational fluid dynamics model in a pneumatically agitated bioreactor

To gain a better understanding of the effects of different model settings, it is necessary to understand the background. When considering hydrodynamics in bioreactors, the proper selection of the discretization scheme, the turbulence model, and the multiphase modeling approach are crucial. Therefore, the underlying concepts are explained in the following (Fig. 1).

### Navier Stokes and Boltzmann equations

Finite Volume computational fluid dynamics (CFD) solving the continuity and Navier Stokes equations operates in a macroscopic framework. Fluids are considered as continua based on averaged properties where each mesh segment contains large amounts of molecules. Changes in the mass of these segments follow the rules of mass conservation and can happen by fluid flow across the boundaries (* A*) of said segments. Given the stationary volume ($\:{V}_{0}$) of the segment and considering the divergence theorem, the continuity equation follows as.

1 $$\:\frac{\partial\:}{\partial\:t}\underset{{V}_{0}}{\overset{}{\int\:}}{\rho\:}_{L}dV=-\underset{\partial\:{V}_{0}}{\overset{}{\oint\:}}{\rho\:}_{L}u\cdot\:dA\:\:\to\:\:\:\frac{\partial\:{\rho\:}_{L}}{\partial\:t}+\nabla\:\cdot\:\left({\rho\:}_{L}u\right)=0,$$

where * t* is the time, $\:{\rho\:}_{L}$ is the liquid density, and * u* is the liquid velocity [27]. For momentum conservation, the transfer by convective and molecular transport, as well as external forces such as gravity have to be considered, leading to

2 $$\:\frac{\partial\:}{\partial\:t}{\rho\:}_{L}u=-\left[\nabla\:\cdot\:{\rho\:}_{L}uu\right]-\nabla\:p-\left[\nabla\:\cdot\:\tau\:\right]+{\rho\:}_{L}g,$$

with * p* being the pressure, $\:\tau\:$ the stress tensor, and * g* the gravitational acceleration.

In contrast to that, the Lattice Boltzmann Method (LBM) acts on a mesoscale level, tracking the evolution of particle distribution functions. Density is generalized as a distribution function $\:f(x,\xi\:,t)$ at position * x*, with velocity $\:\xi\:$ at time * t*, here simply written as * f* for brevity. The change of this distribution function with time can be expressed as

3 $$\:\frac{df}{dt}=\left(\frac{\partial\:f}{\partial\:t}\right)\frac{dt}{dt}+\left(\frac{\partial\:f}{\partial\:x}\right)\frac{dx}{dt}+\left(\frac{\partial\:f}{\partial\:\xi\:}\right)\frac{d\xi\:}{dt}.\:$$

The Boltzmann equation results from Eq. (3) with $\:\frac{dt}{dt}=1,\frac{dx}{dt}=\xi\:,\frac{d\xi\:}{dt}=\frac{F}{{\rho\:}_{L}},\:$and $\:\frac{df}{dt}={\Omega\:}\left(f\right)$ as

4 $$\:\frac{\partial\:f}{\partial\:t}+\xi\:\frac{\partial\:f}{\partial\:x}+\frac{F}{{\rho\:}_{L}}\frac{\partial\:f}{\partial\:\xi\:}={\Omega\:}\left(f\right)$$

[27] where $\:{\Omega\:}\left(f\right)$ is the collision operator defined by Bhatnagar, Gross and Krook as

5 $$\:{\Omega\:}\left(f\right)=\:-\frac{1}{{\tau\:}_{rel}}\left(f-{f}^{eq}\right),$$

with $\:{f}^{eq}$ being the equilibrium distribution. The collision equilibrium is achieved after the relaxation time $\:{\tau\:}_{rel}$ [28]. The equilibrium distribution follows the Maxwellian statistics and is calculated as

6 $$\:{f}^{eq}\left({\rho\:}_{L},{u}_{L},T,\xi\:\right)=\left(\frac{{\rho\:}_{L}}{{\left(2\pi\:RT\right)}^{\frac{d}{2}}}\right){e}^{-\frac{{\left(\xi\:-{u}_{L}\right)}^{2}}{2RT}},$$

Where * d* is the number of spatial dimensions, $\:R={k}_{B}/{m}_{p}$ is the gas constant depending on the Boltzmann constant $\:{k}_{B}$ and mass of the particle $\:{m}_{p}$ and * T* is the temperature [27].

### Discretization scheme

To solve the conservation equations numerically, space and time have to be discretized. Commonly used approaches for discretization apply finite differences (FD), finite elements (FE) and finite volumes (FV). Unstructured grids are mostly implemented with FE and FV methods [29]. In FD, the differential equations are replaced by differences and solved for each grid point [29]. In FE, the domain is split into segments [30] that are used to derive functions for certain properties [31]. In FV, the domain is divided into several control volumes and conservation equations are individually applied [31]. FD, FE and FV have in common that the results of CFD need to be critically checked with respect to the setting of the discretization scheme (e.g., first order upwind, second order upwind, QUICK). Mesh sensitivity studies are crucial to identify mesh densities that allow sufficient local resolution for manageable computation efforts.

On contrary, the domain is spatially discretized by a uniform lattice for LBM calculations preventing complex and time-consuming meshing studies. Nonetheless, demonstrating lattice-size independence remains an essential requirement but is less complex and time-consuming. Then, a distinct set of velocities is chosen to describe the three-dimensional flows, e.g., a set of 19 velocities in three dimensions (D3Q19), which are ((0,0,0), (± 1,0,0), (0,±1,0), (0,0,±1), (± 1,±1,±1)). By correctly scaling the velocities to be proportional to the ratio of grid and time step size, it is ensured that each movement reaches the lattice neighbor per time step [27]. Consequently, at each time step a sequence of a collision/relaxation and a streaming/propagation step is performed. More details on the underlying principles are out of the scope of this study and can be found elsewhere [27].

### Turbulence model: reynolds-averaged Navier-Stokes and large eddy simulations

After choosing conservation balances and discretization schemes, the next step for solving turbulent flows is the choice of a turbulence model. Basic characteristics of turbulent flows are the unsteady, three-dimensional, and vortex-rich motions with fast fluctuations across scales that increase mixing and energy dissipation [32].

For practical applications of CFD, Reynolds-averaged Navier-Stokes (RANS) simulations are often used due to the relatively low computational costs resulting from the lower resolution of turbulence. The latter mirrors the use of averaged flow variables instead of small fluctuations. The averaged values fuel the conservation equations (see Sect. 2.1) [31]. However, an additional closure model for turbulence is necessary. Most well-known ones are the $\:k-\epsilon\:$-model [33], based on the kinetic energy (* k*) and turbulent dissipation ($\:\epsilon\:$), and the $\:k-\omega\:$-model [34]. Here, $\:\omega\:$ is the turbulent dissipation rate [35].

In contrast to RANS, LES resolves large eddies directly filtering out small eddies depending on a chosen threshold. For the latter, suitable sub-grid models are applied [32, 36]. Therein, an additional eddy viscosity ($\:{\nu\:}_{e}$) representing the sub-grid component is introduced as

7 $$\:{\nu\:}_{e}={C}_{s}^{2}{\Delta\:}{x}^{2}\sqrt{S:S},$$

with the Smagorinsky coefficient $\:({C}_{S}$), the grid spacing ($\:{\Delta\:}x$) and the filtered strain rate tensor (* S*) [32]. For further information, the interested reader is referred to [37]. Generally, the filter width $\:{\Delta\:}$ in LES is defined as $\:{\Delta\:}={\left({\Delta\:}x{\Delta\:}y{\Delta\:}z\right)}^{\frac{1}{3}}$ [38]. In our study, LB-LES simulations use a uniform Cartesian grid leading to $\:{\Delta\:}x={\Delta\:}y={\Delta\:}z$ and a filter size equal to the grid spacing in one direction.

### Multiphase modeling: Euler-Euler and Euler-Lagrange

The consideration of multiple phases poses another challenge on CFD. In traditional Euler-Euler (E-E) approaches, the liquid and the gas phase are both considered as continua sharing the same volume. On contrary, bubbles are modelled as point particles in the Euler-Lagrange (E-L) method, considering individual forces and tracking trajectories by integration of Newton’s second law of motion with the Verlet algorithm [39]. Important forces are those for drag, lift, gravity/buoyancy, and added mass. The drag force is a resistance counteracting the bubble movement and can be calculated as

8 $$\:{F}_{D}=\frac{\pi\:}{8}{C}_{D}{d}_{p}^{2}{\rho\:}_{L}\left|{u}_{slip}\right|\left({u}_{slip}\right),$$

with the slip bubble velocity $\:{u}_{slip}$, the bubble diameter $\:{d}_{p}$, and the drag coefficient $\:{C}_{D}$. The drag coefficient was chosen according to Tomiyama as

9 $$\:{C}_{D}=\mathrm{max}\left\{{min}\left[\frac{16}{Re}\left(1+0.15R{e}^{0.687}\right),\frac{48}{Re}\right],\frac{8}{3}\frac{Eo}{Eo+4}\right\},$$

where * Re* is the Reynolds number ($\:Re=\frac{{\rho\:}_{L}{u}_{p,t}{d}_{p}}{\mu\:})$ with the terminal bubble velocity $\:{u}_{p,t}$ and * Eo* is the Eötvös number ($\:Eo=\frac{g\left({\rho\:}_{L}-{\rho\:}_{G}\right){d}_{p}^{2}}{\sigma\:})$ [40].

The lift acts perpendicular to the direction of bubble movement and is caused by a pressure gradient on the sides of bubble due to different liquid velocities [41]. In this study it is implemented according to Saffman [42]

10 $$\:{F}_{L}=1.61\:{d}_{p}^{2}{\rho\:}_{L}\sqrt{\frac{\nu\:}{\left|{\omega\:}_{L}\right|}}\left[{u}_{slip}\times\:{\omega\:}_{L}\right],$$

with the kinematic viscosity $\:\nu\:$ and the fluid vorticity $\:{\omega\:}_{L}$.

The impact of gravity (buoyancy) is calculated as the net effect considering gravitation and the bubble density compared to the liquid density

11 $$\:{F}_{B}=\frac{{\rho\:}_{G}-{\rho\:}_{L}}{{\rho\:}_{G}}g$$

[41].

Finally, the virtual or added mass follows the implementation in the M-Star documentation^1^ and describes the resistance of the accelerated liquid around the moving bubble $\:{V}_{p}$ [41]:

12 $$\:{F}_{VM}=\left(2.1-\frac{0.132}{0.12+{\left(\frac{{u}_{slip}^{2}}{{d}_{p}\frac{d}{dt}{u}_{slip}}\right)}^{2}}\right){V}_{p}{\rho\:}_{L}\left(\frac{{\dot{u}}_{L}-{\dot{u}}_{p}}{2}\right),$$

Compared to E-E, E-L is more computationally demanding but allows a much more detailed analysis of bubble dynamics and therefore might lead to different liquid-bubble interactions [43]. While some authors state that this method is only suitable up to a volume fraction of 10% [9], others do not see this restriction and simulated cases with higher gas fractions [2].

E-L approaches require the knowledge of the so-called “undisturbed flow velocity” [44], i.e. the flow velocity without the influence of particles/bubbles, to calculate the momentum transfer. This quantity is not readily available but has to be estimated. Here, it is approximated as the disturbed flow velocity. However, this is only valid with small errors if the bubble size is much smaller than the mesh size [44]. Some authors claim that a bubble diameter half the size of the mesh is sufficient [45] while others see bubble diameters of up to 10% of the mesh size as the appropriate choice [44].

Beside tracking the fate of individual bubbles in E-L approaches, population balance models (PBM) could be applied to calculate bubble size distributions, also in E-E. Several researchers have coupled CFD with PBM [9, 46–49]. Bubble sizes might be approximated as constant in industrial applications for small initial bubble sizes (about 2 mm) that may be further stabilized by additives and microbial products (ethanol) which inhibit bubble breakage and coalescence [8]. Nevertheless, the influence of the bubble sizes is crucial for accurate flow and mass transfer predictions [47]. The concept of having discrete classes of bubble sizes in PBM might bias the bubble simulations in E-E frameworks, setting arbitrary lower and upper limits, describing the real distribution less accurately than direct tracking and breakage and coalescence considerations for each individual bubble as in the E-L approach [43]. Since the eddy dissipation rate (EDR) can be crucial for bubble breakage [50], the very different ranges for RANS and LES can lead to highly different breakup rates, if the same models are considered [43].

For the free surface tracking, the volume-of-fluid method was used that captures the interface by solving the transport equations of the phases’ volume fraction in the near-surface cells [32, 51].

Mass transfer between the two phases was calculated based on the well-established penetration theory by Higbie [52] as

13 $$\:{k}_{L}=\frac{2}{\pi\:}\sqrt{\frac{{D}_{L}{u}_{slip}}{{d}_{p}}}.$$

This theory corresponds to the surface renewal theory, assuming that liquid elements are in contact with the gas interphase for a limited contact time. As the bubble moves, new elements attach [53].

### Swarm effects

In E-L, coupling forces such as drag ($\:{\overrightarrow{F}}_{D})$, lift ($\:{\overrightarrow{F}}_{L})$, buoyancy ($\:{\overrightarrow{F}}_{B})$, and added mass ($\:{\overrightarrow{F}}_{VM})\:$are typically lumped as ($\:{\overrightarrow{F}}_{f}$) to act on the fluid [26]. Those forces which are calculated based on the diameter of the represented bubbles in the parcel are scaled by the parcel size to the power of the scaling exponent * n* ($\:{N}_{p}^{n}$), where * n* is a parameter between 0 and 1 (by default 0.5 in M-Star CFD).

14 $$\:{\overrightarrow{F}}_{f}=-{\sum\:}_{p}^{p\in\:j}{N}_{p}^{n}\left({\overrightarrow{F}}_{D,p}+{\overrightarrow{F}}_{L,p}+{\overrightarrow{F}}_{B,p}+{\overrightarrow{F}}_{VM,p}\right)$$

Reducing the force coupling between the gas and the liquid phase by an exponent (* n*) represents swarm effects that apply if many bubbles interact. Swarm effects in force formulations are widely discussed (e.g [54–56]), and might occur especially in industrial-scale applications with high gassing rates. The parcel size strongly influences local gas holdups which are usually considered in drag force corrections for swarm effects [56]. High local gas holdups were further shown to reduce the average vertical velocity experimentally [57]. Impacts include rheology changes, shielding effects by leading or trailing particles, as well as returning flows by neighboring particles [58, 59]. In the present study, viscosity and hydrostatic changes are likely but bubble diameters are too small for creating shielding effects.

In LB-LES, the influence of larger parcel sizes on the predicted mean liquid velocity can be described, analogous to the swarm effect. With fitting parameters $\:\alpha\:$ and $\:\beta\:$, a correlation of the mean liquid velocity ($\:{\stackrel{-}{u}}_{L}$) depending on the parcel size can be given as

15 $$\:\frac{{\dot{V}}_{L}}{{V}_{L}}=\alpha\:\cdot\:{N}_{p}^{\beta\:}\:\:\mathrm{w}\mathrm{i}\mathrm{t}\mathrm{h}\:\:\:{\dot{V}}_{L}={\stackrel{-}{u}}_{L}\cdot\:{A}_{R}.\:$$

By relating the mean flow velocity to the reactor’s cross section area ($\:{A}_{R}$) and the liquid volume in the tank ($\:{V}_{L}$), scales are considered. Here, a similar scaling with $\:{N}_{p}$ and an exponent is applied as in the force coupling. This approach allows to determine the liquid velocity with a parcel size ($\:{N}_{p}$) of 1 if values for other parcel sizes are available. The parameter $\:\alpha\:$ represents $\:{\dot{V}}_{L}/{V}_{L}$ for the parcel size of 1 and can be determined by parameter fitting.

### Geometry and model parameters

**Fig. 2:**
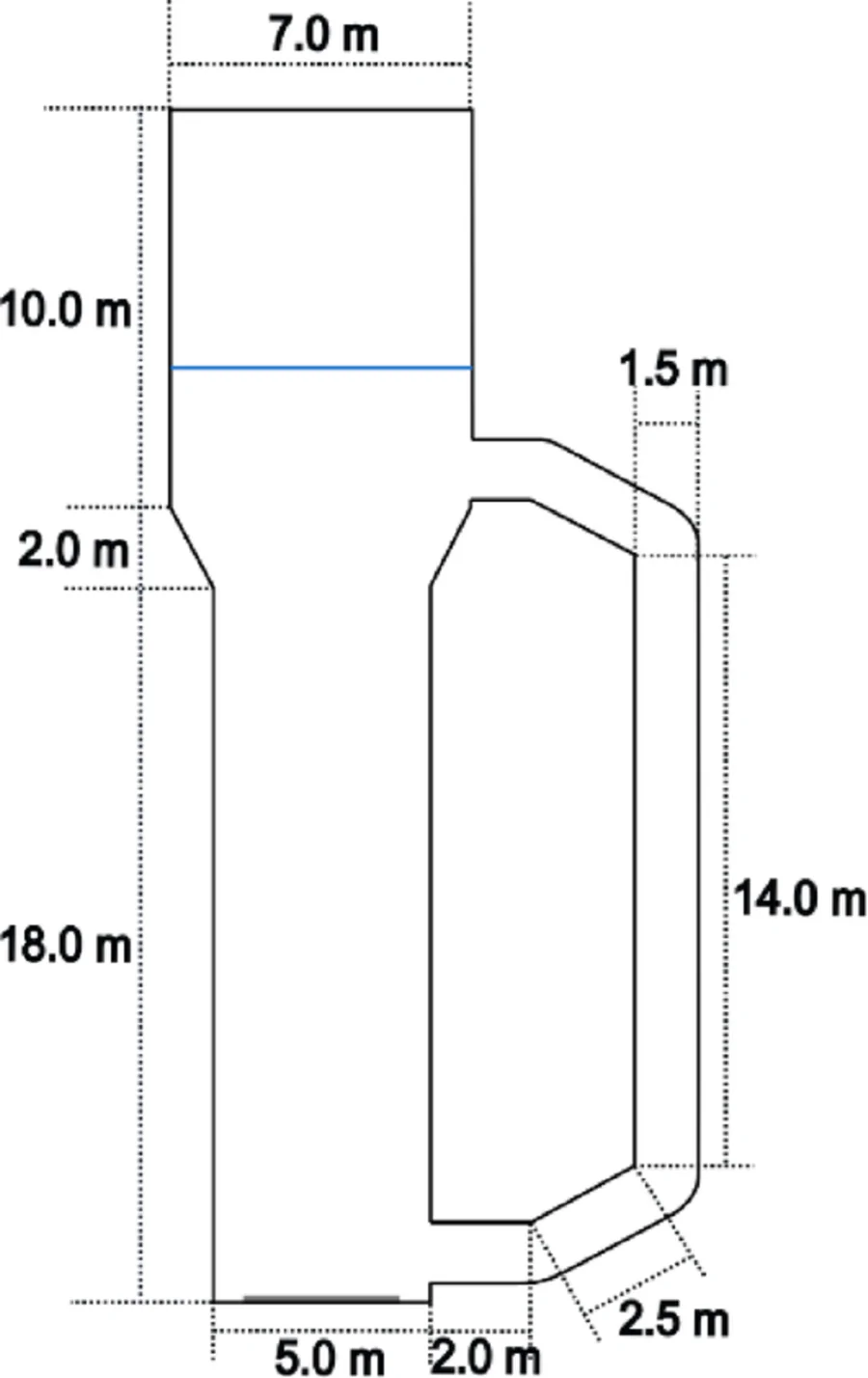
Geometry of external loop airlift reactor model

For the simulation studies, an external loop airlift reactor (EL-ALR) (see Fig. 2; Table 2) from the work of [8] was simulated using LB-LES with the software M-Star CFD 3.9^2^. To be able to simulate the large 800 m³ tank, the parcel approach had to be applied, grouping bubbles to parcels. Hence, a collection of bubbles is tracked instead of an individual bubble. Consequently, parcel/liquid interactions scale with number of bubbles per parcel, i.e., the parcel size [26]. Recently, large parcel sizes of 500 bubbles per parcel were used [2] to make the simulation of an industrial-scale reactor manageable. This pragmatic decision also mirrors findings of Thomas et al. [26] indicating that the impact of the parcel size is minimal in STRs as long as a statistically relevant number of parcels is tracked. However, this was only shown in a stirred reactor, where the liquid movement is dominated by the mechanical rotation. In the case of pneumatically agitated bioreactors, the bubble-liquid interaction is the main driver of movement, making further investigations on the accuracy with larger parcel sizes necessary.

Since more than 9 billion bubbles are present in the tank, this number cannot be individually tracked. For a parcel size of 30, the simulation ran out of memory after 160 s due to the increasing number of bubbles on a system with two linked 48 GB GPUs (NVIDIA RTX A6000). Therefore, the parcel size of 50 was chosen as the lowest number. Additionally, larger parcel sizes of 150 and 300 were tested to increase simulation speed with the same amount of ‘simulated’ bubbles. All runs were conducted for 400 s to get to a (transient) steady state. Mesh and time step independence studies were conducted with a parcel size of 150 and the results are given in the supporting material. There vertical velocity fields are compared to demonstrate the feasibility of the approach.

To be able to compare with a parcel size of one, the pilot-scale EL-ALR model by [60] was used and the study was conducted in pilot-scale with parcel sizes of 1, 50, 150, and 300. A correlation based on power law ($\:y=\alpha\:\cdot\:{x}^{\beta\:}$) was fitted to the data based on the MATLAB Curve Fitting Toolbox^3^. To consider different scales, the mean velocity ($\:\stackrel{-}{u})$ was transformed to liquid flow per volume ($\:\stackrel{-}{u}\cdot\:{A}_{R}\backslash\:{V}_{L}$). For industrial-scale, parameter $\:\beta\:$ was fitted based on the confidence interval of the determined $\:\beta\:$ in pilot-scale (Eq. 13). Validation of the final model settings was performed based on a process by [7] with available experimental measurements in Sect. 3.2.

When comparing results of LB-LES with FV-RANS, differencing of the flow fields was chosen for visualization purposes to highlight deviations and similarities. In order to make the results of the two approaches more comparable, both results can be time-averaged. Thereby, transient turbulent structures are averaged out.

**Table 2:** Model settings for the LB-LES hydrodynamic model of the external loop airlift reactor, largely derived from [8]

Properties	Conditions	Units	References
*Model settings*			
Gas inlet	Mass flow feed: 2.11 (1.27e + 8)	kg/s (bubbles/s)	[8] (considering hydrostatic pressure)
	Composition: CO: 50; N2: 50	vol%	[8]
	Injection type: injection on sparger surface		
Outlet	Free Surface		
Boundary conditions	No-slip (walls) Free-slip (reactor outlet) Mid-way bounce back approach (bubbles)		[61] See supporting material 1
Bubble size at reference pressure	3	mm	[8]
Bubble-bubble interaction	No interaction		
Bubble breakage and coalescence	Not considered		[8]
Multiphase modeling	Euler-Lagrange		
Turbulence	LES, Smagorinsky subgrid-model		[36]
Smagorinsky coefficient	0.1		[62]
Phase interactions	Gravity		
	Drag		[40]
	Virtual mass		[63]
	Lift		[42]
	Additional force*		
Fluid-bubble coupling	Newton’s third law		
Parcel size	30, 50, 150, 300		
Time step size	0.000175	s	
Total mesh size	60.1 M		
Reference pressure	101,325	Pa	
Reference temperature	273.15	K	
*Fluid properties*			
Density liquid	998	kg m^− 3^	[8]
Viscosity liquid	1.002e-6	m^2^ s^− 1^	[8]
Surface tension	0.0522	N m^− 1^	[64]
Density gas	Ideal gas law		
Liquid volume	approx. 570	m^3^	[8]
*Scalar coupling (CO)*			
Mass transfer coefficient	Higbie		[52]
Henry coefficient	0.0212		[65]
Biomass concentration	15	g L^− 1^	in the range of [66]
Molar volume	Ideal gas law		

*additional force in certain areas ($\:{y}_{p}>18.6\:\&\&\:{y}_{p}<22\:\&\&\:{x}_{p}>3.4$ or $\:{y}_{p}<3.5\:\&\&\:{x}_{p}>2.5)$ if the bubble volume fraction exceeded a value of one that was introduced to prevent this unrealistic bubble accumulation (push downwards and acceleration in x-direction to follow the flow instead of being stuck in a region that is already filled with bubbles, amplified by an acceleration factor identified by trial-and-error, $\:{F}_{add,X}=15000\cdot\:{u}_{x,p}\cdot\:{V}_{p}\cdot\:{\rho\:}_{p},\:{F}_{add}$,$Y=-15000\cdot\:{u}_{y,p}\cdot\:{V}_{p}\cdot\:{\rho\:}_{p}$), for further information see Supporting material 1

## Results and discussion

### Influence of parcel size on model predictions

**Fig. 3:**
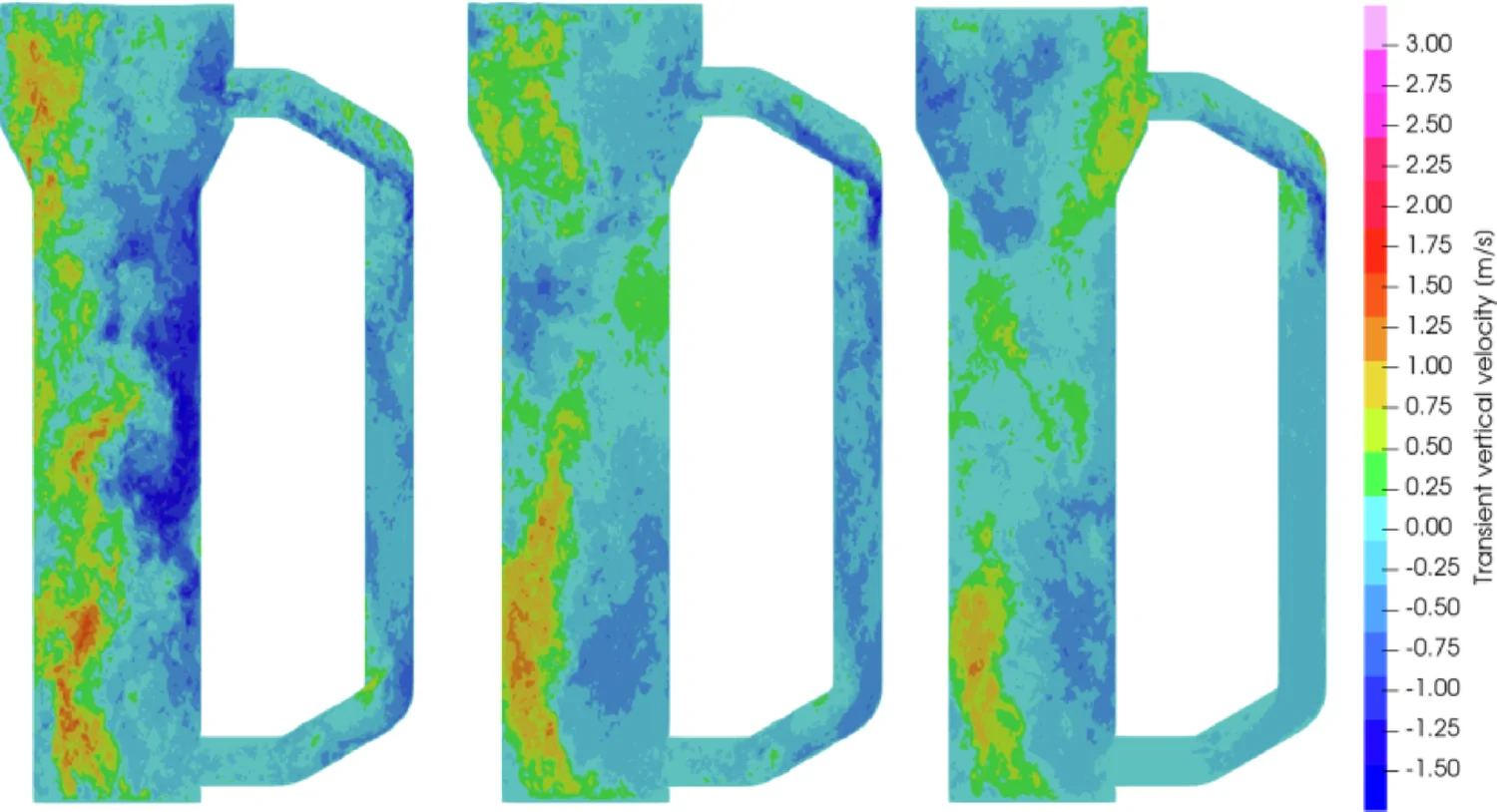
Visualization of snapshots of the transient vertical velocity fields in the industrial-scale external loop airlift reactor simulated with LB-LES for parcel sizes of 50 (left), 150 (middle), and 300 (right) after reaching a pseudo-steady state

To investigate the impact of parcel size on the simulated flow fields in a pneumatically agitated bioreactor, the system was modeled and simulated with parcel sizes of 50, 150, and 300 for 400 s each. The comparison of the transient vertical flow fields (Fig. 3) revealed different maximal and minimal velocities. The flow velocity inside the downcomer was reduced with a parcel size of 300 compared to the other cases. The highest negative velocity with lowest parcel size was found in the right part of the riser, counteracting the high velocities closer to the left wall. Deviating flow distributions, such as the higher velocity at the entry of the downcomer for parcel size 300 may be attributed to the transient nature of the simulations. Table 3; Fig. 3 show that maximal and mean velocities generally decrease with increasing parcel sizes. Likely, the trend continues for parcel size below the technical memory limit of 50. Hence, liquid velocities are likely to be underestimated with larger parcel sizes.

**Table 3:** Model readouts and time necessary for simulations of different parcel sizes in an industrial-scale EL-ALR

Parcel size	50	150	300
Global mean/max liquid velocity [m s^− 1^]	0.5441 ± 0.0265/ 2.8736 ± 0.4877	0.3993 ± 0.0129/ 2.0351 ± 0.1997	0.3540 ± 0.0067/ 1.7369 ± 0.1698
k_L_a value Higbie [h^− 1^]	614.16 ± 15.12	657.36 ± 3.24	680.40 ± 7.20
k_L_ Higbie [m s^− 1^]	6.108e-4 ± 0.116e-4	6.078e-4 ± 0.115e-4	6.089e-4 ± 0.115e-4
Interfacial area a [m^− 1^]	279.30 ± 7.20	300.43 ± 2.25	310.42 ± 3.21
Cumulative wall time to simulate 10 s [h]	9.03	2.99	1.68

An explanation for the underestimated liquid velocities with increasing parcel size could be the non-sufficient scaling of interacting forces (see Eq. 14).

#### The scaling factor as a proxy for considering swarm effects

After introducing the parcel size, the scaling factor ($\:{N}_{p}^{n}$) intrinsically reduces the impact of bubble-to-liquid momentum transfer and centers it to fewer computational cells finally creating divergences. In essence, the parcel approach creates local ‘swarms of bubbles’. The phenomenon already exists in the bioreactor as numerical force-scaling effects but the parcel approach intensifies it. True physical swarm effects are known to reduce the bubble-to-liquid force, which in analogy also applies while scaling the forces according to the parcel size (see Eq. 14). Therefore, lower velocities determined with larger parcel size might even be more realistic than values with parcel size 1 which do not consider swarm effects. However, experimental validation of this hypothesis is necessary which is out of the scope of this study.

When considering the sum of all forces without scaling (* n=1*), no stable simulations are achievable. The forces applied on individual cells are too large. Yang et al. [56] list approaches to include swarm effects on the drag force that are mainly based on the local gas fraction. In turn, it is strongly influenced by the parcel size.

Overall, the links between parcel size, gas fraction, and momentum transfer are complex. The identification of sound exponents mimicking the realistic swarm physics would require further (experimental) investigations under large-scale conditions.

**Fig. 4:**
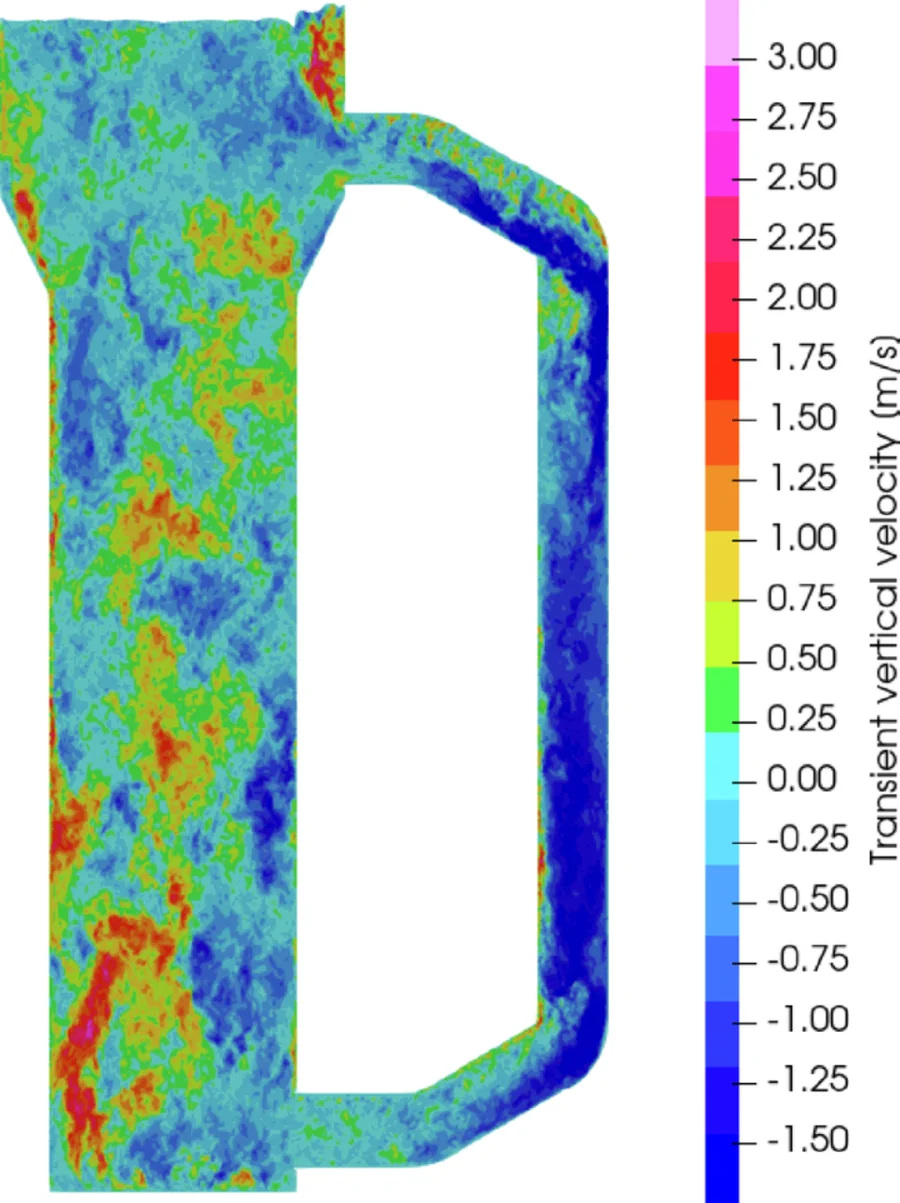
Transient vertical velocity field of the industrial-scale EL-ALR with a third law scaling exponent of 0.9 and parcel size of 150

Increasing the default value of * n=0.5* to * 0.9* reduced the swarm impact. As a result, higher velocities were observed in the tank (see Fig. 4).

Increasing parcel sizes may create other deviations besides inaccuracies of force scaling. In other applications, authors already pointed out that underestimations might be caused by differences of local bubble distributions. Using parcels causes the spatial distributions of bubbles to deviate from that obtained with individual Lagrangian particles [25]. The first causes locally high liquid accelerations that lead to large deviations between disturbed and undisturbed liquid velocities. For the sake of simplicity, the latter are typically used as proxy for the undisturbed velocities while balancing forces [44]. Hence, the larger the parcel size, the stronger the biasing impact on the approximated undisturbed velocities. This impacts the drag force which depends on the undisturbed velocity and the particle/bubble velocity [44]. Resulting drag forces might be lower which in turn leads to less acceleration of the surrounding liquid finally underestimating the liquid velocity.

**Fig. 5:**
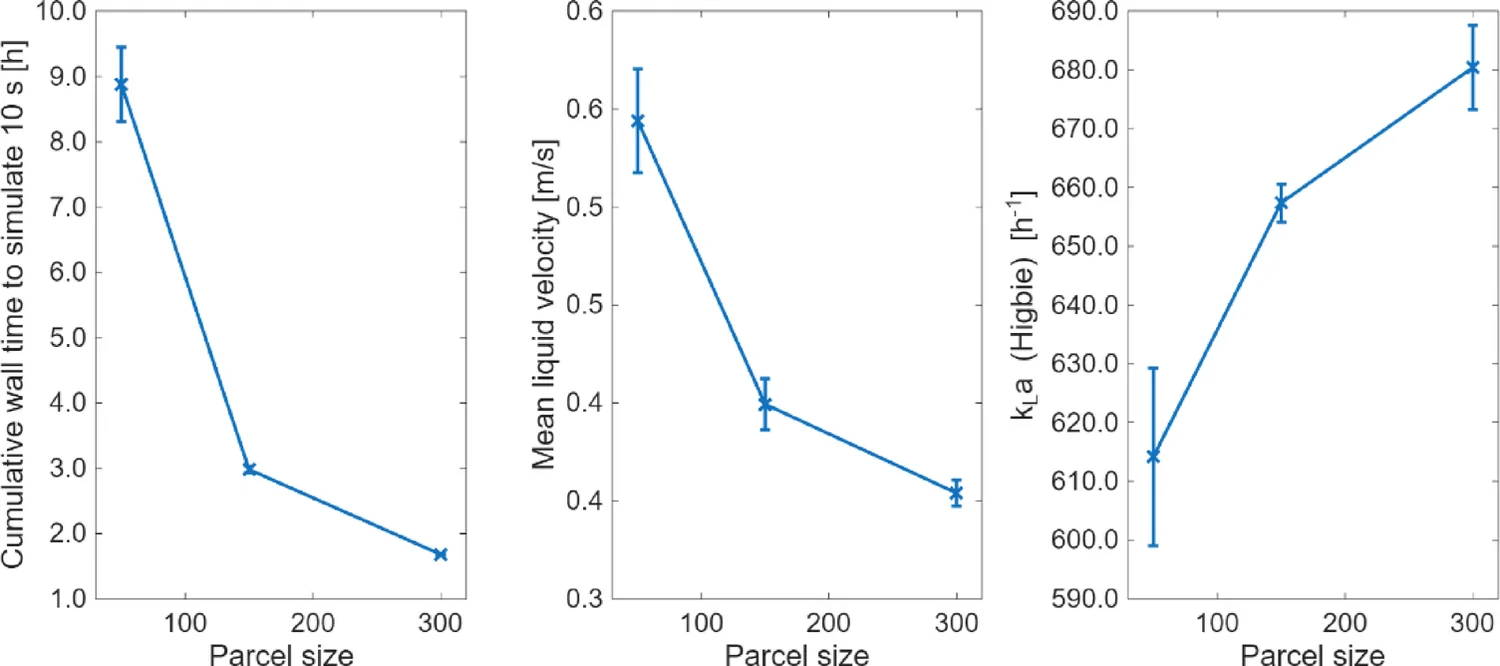
Change of cumulative wall time to simulate 10 s (left), mean liquid velocity (middle), and mass transfer coefficient (k_L_a) (right) based on Higbie with parcel size. Values were averaged over the last 80 s and the standard deviation over that time is given as error bars

Besides the impact of parcel size on the mean liquid velocity, the influence on computational time and mass transfer coefficient (*k*_*L*_*a*) was studied, too (Fig. 5). From the perspective of practicability, the importance of the parcel approach gets obvious when investigating the savings of computational time. With a parcel size of 50, simulating 10 s of flow takes more than 9 h on the two 48 GB GPUs. In other words, simulating 100 s (which is still not sufficient for any statements about the process in this scale) would take almost 4 days. While this may still be acceptable for research purposes, expectations of industrially relevant applications would not be met sufficiently. However, compared to parcel size 50, parcel sizes of 150 and 300 require only a third and a fifth of the computational time, respectively. Notably, larger tank sizes or more intense aeration conditions may ask for even larger parcel numbers [2]. However, the lower limit is also constrained by the technical memory installed. Already parcel sizes of 30 caused ‘out-of-memory’ error which intrinsically set a lower bound for the minimum necessary number of parcel size. As a consequence, underestimating liquid velocities intrinsically shall be accepted if industrial scale bioreactors should be studied in reasonable computation time.

Apart from impacting liquid velocities, parcel sizes also affect the mass transfer coefficient *k*_*L*_*a* as indicated in Fig. 5. Again, the ‘artificially’ low velocity difference between the uprising bubbles and the surrounding fluid is the reason: Now, bubbles stay longer in the bioreactor which finally increases the specific interfacial area (*a*) (Table 3). At the same time, the impact on the slip velocity is rather low causing the mass transfer constant (*k*_*L*_) to remain. As a consequence, the product *k*_*L*_*a* rises with parcel size from 612 h^− 1^ up to 679 h^− 1^.

Motivated by the observations of large-scale simulations, the pilot scale setting was revisited as it offers the possibility to apply LB-LES even with the parcel size of 1. Notably, the pilot EL-ALR is a downscaled version of the large-scale EL-ALR as published in [60]. Regarding the pilot-scale, parcel sizes of 1, 50, 150, and 300 cause the same trend as observed for the large scale (see Tables 3 and 4). With a scaling exponent of 0.5, mean and maximal liquid velocities decrease notably with increasing parcel sizes. At the same time, * a* and thereby $\:{k}_{L}a$ increase. While large savings in computational time are observed from parcel size 1 to 50, savings are getting less when increasing parcel sizes to 150 and 300.

However, observations with a scaling exponent of 1 are much different: Liquid velocities almost remain irrespective of the parcel size. The interfacial area * a* decreases which in turn reduces $\:{k}_{L}a$. Hence, considering swarm effects based on the parcel approach force scaling (*n* = 0.5) leads to different results than ignoring swarm effects (*n* = 1). For the latter, bubble residence times are lower, possibly due to the inhomogeneous distribution of bubbles in the tank.

**Fig. 6:**
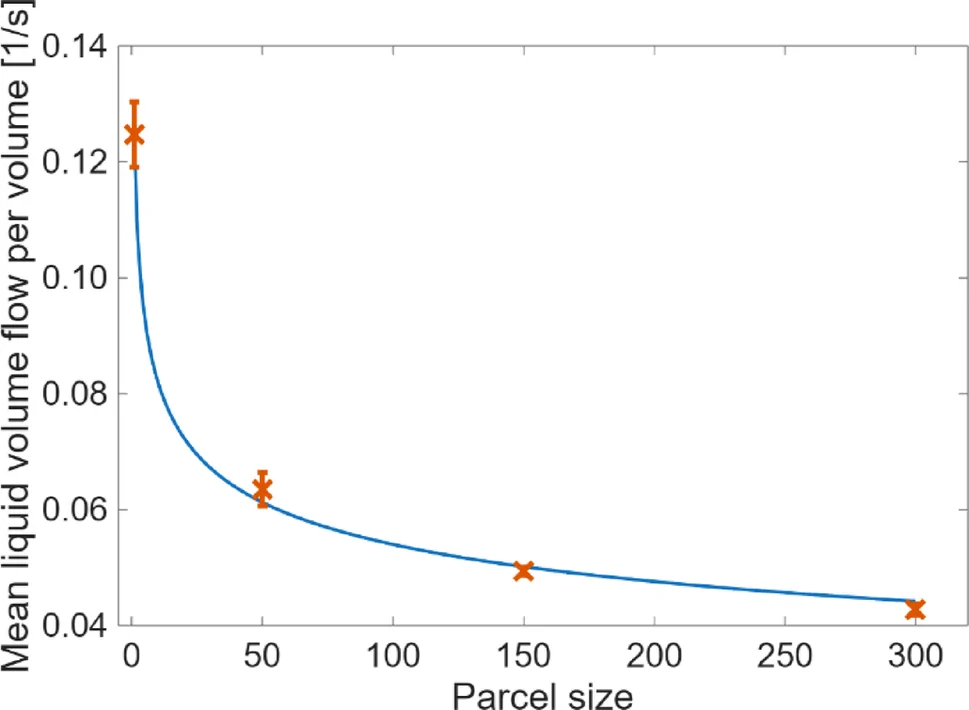
Fit of the correlation in Equation (15) to the simulation data (parcel size vs mean liquid velocity) in the pilot-scale EL-ALR *α = 0.1250*$~\left[ {0.1166,0.1334} \right]$, *β = - 0.1823*$\left[ { - 0.2066, - 0.1581} \right]$,$~{R^2} = 0.9981$

The consideration of the reference case with parcel size 1 now allows to identify a correlation function given in Eq. (15). The simulation results are shown in Table 4 and the correlation function fit in Fig. 6. Compared to the industrial-scale, swarm effects might be neglectable in pilot-scale due to the lower number of bubbles per cell.

**Table 4:** Model readouts and time necessary for simulations of different parcel sizes in a pilot-scale EL-ALR with third law coupling exponent of 0.5 (M-Star CFD default value) and 1

Parcel size	1	50	150	300
*Third law coupling exponent 0.5*				
Global mean/max liquid velocity [ms^− 1^]	0.3078 ± 0.0140/1.3653 ± 0.1055	0.1568 ± 0.0072/0.6486 ± 0.0394	0.1219 ± 0.0017/0.5248 ± 0.0335	0.1056 ± 0.0024/0.4703 ± 0.0388
k_L_ a value Higbie [h^− 1^]	302.49 ± 5.27	317.02 ± 7.90	328.42 ± 5.35	357.91 ± 4.59
k_L_ Higbie [ms^− 1^]	6.72e-4	6.76e-4	6.65e-4	6.41e-4
Interfacial area a [m^− 1^]	125.24 ± 2.27	130.24 ± 3.28	137.16 ± 2.19	155.13 ± 2.04
Cumulative wall time to simulate 10 s [h]	2.003 ± 0.048	0.318 ± 0.003	0.308 ± 0.003	0.303 ± 0.002
*Third law coupling exponent 1*				
Global mean/max liquid velocity [ms^− 1^]	0.3078 ± 0.0140/1.3653 ± 0.1055	0.3045 ± 0.0078/1.3671 ± 0.1037	0.3063 ± 0.0088/1.3725 ± 0.1092	0.3071 ± 0.0131/1.4453 ± 0.1190
k_L_ a value Higbie [h^− 1^]	302.49 ± 5.27	293.30 ± 3.46	280.18 ± 4.89	262.89 ± 2.70
k_L_ Higbie [ms^− 1^]	6.72e-4	6.83e-4	6.82e-4	
Interfacial area a [m^− 1^]	125.24 ± 2.27	119.36 ± 1.37	114.84 ± 2.13	109.86 ± 1.26
Cumulative wall time to simulate 10 s [h]	2.003 ± 0.048	0.446 ± 0.098	0.318 ± 0.002	0.299 ± 0.003

Fitting the same correlation to the industrial-scale data, the related mean (reference) velocity can be predicted. Therefore, $\:\beta\:$ is kept in the confidence interval determined for pilot scale, while $\:\alpha\:$, which represents the reference velocity at parcel size one, is fitted ($\:\alpha\:=0.0361\:\left[-\mathrm{0.1198,0.1921}\right]$, $\:\beta\:=-0.1823\:\left[-\mathrm{1.0942,0.7296}\right]$, $\:{R}^{2}=0.9165$). In this way, the mean (reference) velocity of 0.92 m/s for parcel size one is predicted for the industrial scale. This shows that even low parcel sizes of 50 lead to high deviations in the predicted liquid velocities when considering a third law scaling exponent of 0.5. Likely, this mirrors the impact of swarm effects in the industrial scale.

If the scaling factor is set to 1 (pilot-scale) or to 0.9 (industrial-scale, to consider the observed limits of numerical stability), the liquid velocity is predicted much higher and therefore closer to the estimation with parcel size one. One notable difference that persists is the decreasing * a* and likewise reduced $\:{k}_{L}a$. Apparently, the global * a* is underpredicted by using the parcel approach.

Depending on the available resources, the available time for the simulation, and the purpose of the model, a compromise between the accuracy and the computational demand has to be found. In pilot scale, the largest deviations were found when increasing the parcel size from one to 50 (see Fig. 6). Therefore, moderate parcel sizes already lead to highly deviating results compared to parcel size 1. Those are stronger for parcel size of 150 that offered large savings in computation time for the industrial application. Hence, using the parcel approach for industrial scale inevitably leads to deviations compared to parcel size 1. However, they at least partially mirror the impact of bubble swarm physics that are otherwise neglected. To select the “right” parcel size, experimental evidence is required, for example on mean liquid velocities and gas holdups.

Another alternative to the third law two-way coupling between bubbles and liquid might be the density coupling. Density coupling assumes buoyancy to be the dominating force, only applying its effect to the liquid as a local body force. However, the effect of drag on the liquid is in principle not negligible in pneumatically agitated reactors. While this approach might be applicable at pilot scale where drag is weaker, it is not physically sound in the drag-dominated industrial-scale airlift reactor.

### Validation of model settings based on experimental data

Since no experimental data for the showcasing process was available, the work by [7] was used for validating the model settings. The same number of grid points along the x-axis, Courant number, and parcel size (here 150) were applied. For validation purposes, two different scaling exponents (see Eq. 14) were tested, 0.5 and 1. In experiments, the authors determined a mixing time of around 150 s measured by probes [7]. Simulations of our model delivered different mixing times depending on the manner of injection and the determination method. With a force scaling exponent (* n*) of one, the free surface was not stable, leading to unrealistic results and really low mixing times. Applying the same injection volume as the experimentalists with a scaling exponent of 0.5, mixing times of 145 and 155 s were found when allocating standard deviations of all tank segments after the two subsequent pulses. They were injected just below the liquid surface in the middle of the tank with a 10 s interval between them. Besides, mixing times of 135 s were detected for a simulated probe fixed at 5.85 m, 10 cm away from the wall. If the injection spot shifted from the middle to one side, mixing time decreased slightly to 130 s for both, the integral mixing time and the local mixing time at a probe.

Summarizing, we concluded a sufficient agreement between simulation and experiments with deviations of about 10% that may reflect measurement and modelling errors. Notably, the error is smaller than the deviations of the mean liquid velocity that we have observed as an impact of increasing parcel size larger than 50 (27%).

Further, a force scaling exponent of 0.5 was validated. However, the mixing time only represents a global variable, that does not give evidence on local deviations between the experimental and simulation results. As local variables are used for model comparison, this is a major limitation of the present study. However, acquiring spatially resolved measurements at multiple locations within an industrial-scale system is associated with considerable risk and substantial costs, rendering such approaches impractical for industrial operation. Consequently, validation based on the mixing time provides at least partial support for the predictive capability of the model. Future advances in sensor technology may enable more comprehensive acquisition of local validation data and thereby improve model verification.

### Comparing finite volume Reynolds-averaged Navier-Stokes with lattice Boltzmann large eddy simulations

After validating the model with a parcel size of 150, two different multi-phase models were compared: FV-RANS using E-E and LB-LES with E-L multiphase. The aim was to identify which method suits better for large-scale simulations of pneumatically agitated bioreactors. Advantages and disadvantages of either approach should be identified.

Some studies have already examined differences between FV-RANS and LB-LES for specific applications. Haringa [21] simulated an industrial-scale STR with LB-LES and compared the results with FV-RANS simulations. The author stated that RANS cannot capture details of the dynamic behavior, e.g., neglecting oscillations that affect mixing [13]. On contrary, LB-LES predicted stronger axial convection and asymmetry in the flow field leading to diverging regimes that mirror flow dynamics more realistically. While differences in mixing time were minor, lifelines showed more variance in LB-LES compared to FV-RANS due to the turbulence handling [21]. At the same time, LB-LES benefited from reduced computational burden thanks to its high degree of parallelization.

The current study poses different challenges as bubble column flows are not dominated by mechanical stirring. Additionally, the impact of parcel size needs to be considered which is only of minor importance in stirred tanks [26]. More recently, Mast & Takors [43] compared the flow predictions for BCRs with RANS and LB-LES in small-scale tanks. The authors observed higher eddy dissipation rates (EDRs) in the LB-LES since time averaging decreased the related values using FV-RANS. The study showed what adaptations are necessary to transfer bubble breakup models between the two approaches [43]. Notably, parcel sizing was not applied thanks to the small reactor volume analyzed. Other authors have compared RANS and LES mainly for gaseous systems [67, 68], highlighting LES’ superior predictive power for dynamic phenomena and improved turbulence capture capabilities.

In this study, industrial-scale EL-ALR, LB-LES results were compared with FV-RANS simulations by Puiman et al. [8]. As the latter could not consider swarm effects, LB-LES simulations were performed with the third law scaling exponent of 0.9. Please note, that the exponent 1 caused numerical instability. Choosing the scaling factor 0.9 implies that only minor swarm effects are included in the LB-LES simulations.

**Fig. 7:**
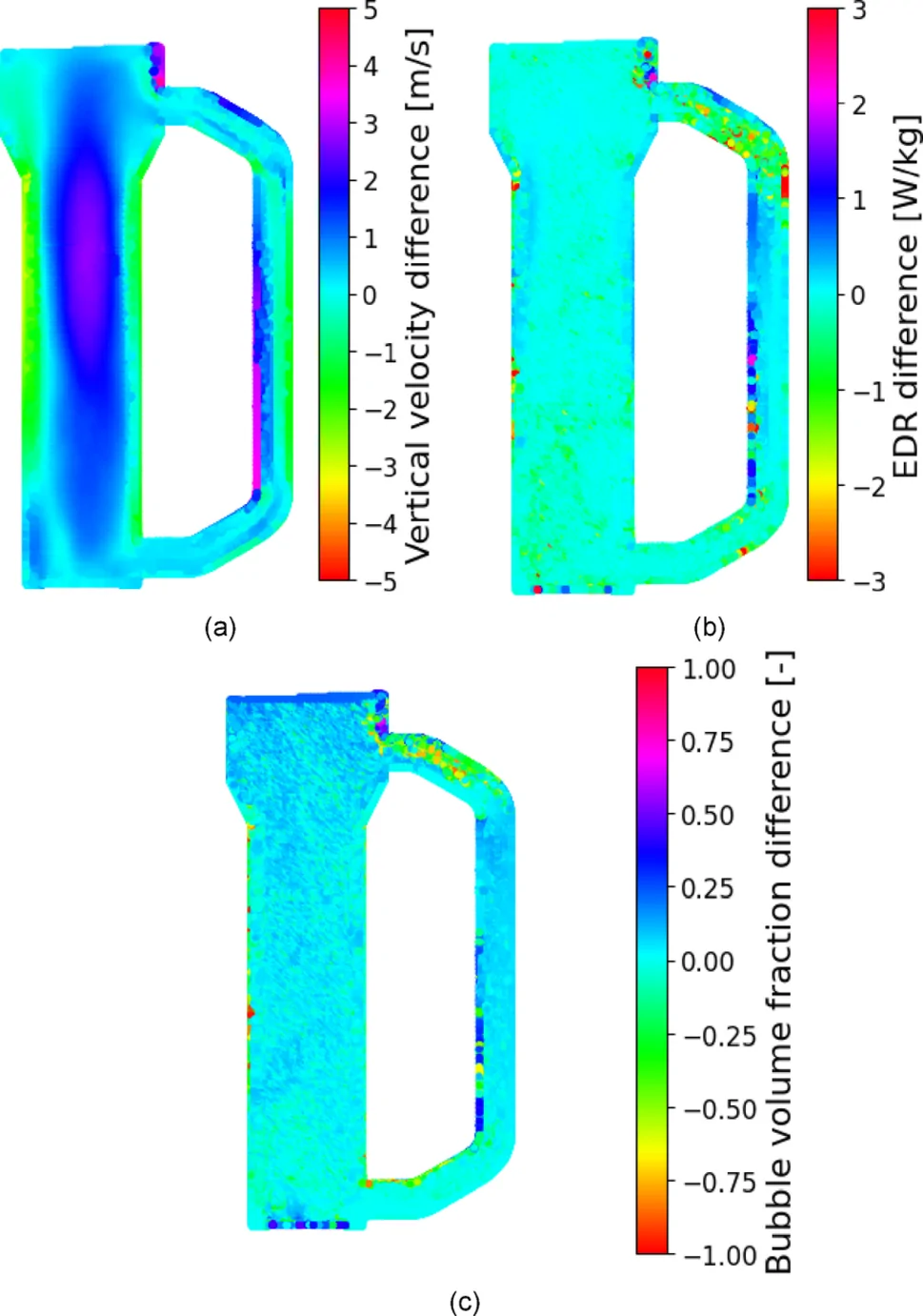
Differential plots: Difference between **a** the time-averaged steady-state vertical velocity flow field, **b** snapshots of eddy dissipation rates (EDR), and **c** the bubble volume fractions calculated with FV-RANS by [8] and the time-averaged transient flow field generated with LB-LES simulations with a third law coupling exponent of 0.9 in this study for an industrial-scale external loop airlift reactor. Positive values indicate higher FV-RANS values, negative values indicate higher LB-LES values

The comparison of time-averaged LB-LES and FV-RANS vertical velocity fields revealed significant deviations (Fig. 7). Maximum velocities in LB-LES (maximal vertical velocity of 1.88 m/s) were much lower than in FV-RANS (maximal vertical velocity up to 5.76 m/s). The really high velocity above the downcomer entry in the FV-RANS may be unrealistically high, possibly caused by escaping bubbles that then reach the surface. This may be attributed to the E-E bubble treatment, neglecting individual bubble physics. The mean absolute vertical velocities are more comparable but still deviate (0.78 m/s in FV-RANS, 0.52 m/s in LB-LES). This is possibly caused by the parcel-based approach. Maximal downflow velocities on the other side were highly comparable (−2.12 m/s in FV-RANS, −2.28 m/s in LB-LES) indicating that the flow in the downcomer was predicted fairly similar. Even with the smallest parcel size (50) or scaling exponent 0.9, LB-LES could not reproduce the highest velocity observed by Puiman et al. [8]. Nevertheless, the overall velocity magnitudes agreed reasonably well.

The position of maximum time-averaged velocity also differed: LB-LES predicted it closer to the bottom of the reactor where downflow from the downcomer interacts with the gas sparger stream. FV-RANS located it higher in the riser, where bubbles can rise freely [8]. The upward flow was concentrated in the riser center in the FV-RANS results while it remains pushed to the wall opposite to the downcomer outlet in the LB-LES simulations.

In the downcomer, LB-LES did not reveal reversed upwards flow as the RANS results showed, leading to large deviations there. The downflow velocities on the other side were comparable in the two cases. Due to the time-averaging of the LB-LES results, the two approaches were more comparable. Considering the transient results by LB-LES, more turbulence than in the RANS approach is captured which is averaged out here.

Despite local differences in velocity magnitude and turbulence, both models reproduced similar overall flow structures: flow towards the tank wall opposite the downcomer outlet, circulation back down close to the downcomer outlet, the high velocity located in the middle of the downcomer, recirculation in the head part, and high velocity flow in the entry of the downcomer tube. In these areas, the deviations were minor (Fig. 7a).

A subsequent comparison of the eddy dissipation rates (EDRs) (Fig. 7b) and bubble volume fractions (Fig. 7c) revealed generally consistent trends between the two modelling approaches. In the bulk flow region on a single 2D-plane, EDRs were nearly identical with a slightly lower prediction by FV-RANS. The FV-RANS simulations, however, predicted locally elevated EDRs (difference > 0) above the downcomer entry, along the straight section of the downcomer, and directly at the sparger. In contrast, the LB-LES results showed higher EDRs at the wall opposite the downcomer outlet, directly above the sparger, and in the bends of the external loop. These values were surprisingly similar compared to literature comparisons of EDRs between the two approaches [43]. RANS generally tends to underpredict EDR [69], which in this case was only apparent in certain regions and not clearly visible on the visualized slice. However, the integral values reveal this underprediction with RANS yielding 25.99 W m^3^ kg^− 1^ for RANS compared to 86.28 W m^3^ kg^− 1^ for LES. One possible reason for the apparent similarity on the slice is the slight swarm effects in the LB-LES simulations that may locally dampen turbulence and reduce differences relative to RANS predictions. It should also be noted that LES values are highly transient and spatially inhomogeneous, which could explain low values on individual slices at certain time points.

The observed similarities suggest that optimizations based on flow structure, such as internal geometries proposed by Bokelmann et al. [60] would lead to comparable results irrespective of the modelling approach. Deviations might be mainly caused by the different considerations of turbulence. The LB-LES simulations considered more detailed dynamics, leading to highly transient structures.

Another aspect concerns the treatment of the multiphase flow. E–E approaches are prone to numerical diffusion, whereas E–L approaches may underpredict gas holdup and fail to capture free surface dynamics [70, 71]. In some cases, E–E simulations yielded velocity fields closer to experimental data, while E–L underestimated velocities at high gas flow rates due to missing bubble–bubble interactions [70]. In the present study, the E-L method resulted in the overall gas holdup of 0.10 compared to 0.13 of the RANS study [8]. Since the RANS results were validated by comparison with empirical correlations for the gas holdup (calculated values of 0.11 to 0.15 for different correlations) [8], this further indicates validity of the model in the current study.

For the comparison of the local bubble volume fractions, minor corrections were required to the LB-LES results. Since bubbles are represented as massless point particles in E-L, they tend to accumulate, especially when using the parcel approach, occasionally exceeding the physical maximum of 1 (see Supporting material 1). To enable meaningful comparison, the values were capped at this limit. This issue was not observed in the FV-RANS results. This unphysical phenomenon represents a major limitation of the LB-LES results so far and should be considered more carefully in future implementations of the method. Overall, the spatial distribution trends of bubble volume fraction were comparable across the two approaches. Notably, FV-RANS predicted higher bubble volume fractions near the liquid surface, whereas LB-LES indicated elevated bubble fractions at the downcomer entry.

These differences could be attributed to the distinct ways the models handle turbulence–bubble interactions: FV-RANS may promote more uniform vertical mixing due to modelled turbulence dissipation, while LB-LES resolves finer-scale turbulent structures that can trap bubbles locally, particularly at flow entry regions or near geometric features.

Similarly large deviations were found in the work by Ashraf Ali & Pushpavanam [68], where locally maximal values of 0.2 for E-L compared to 0.35 for E-E were found. This finding is particularly interesting when considering that LB-LES predicted higher interfacial areas in combination with lower velocities in the parcel size study (Sect. 3.1). Higher velocities in RANS likely caused longer bubble residence times and higher overall holdup by pushing more bubbles into the downcomer. This can be observed in the straight section of the difference plot in Fig. 7(c). Nevertheless, the predicted mass transfer coefficient ($\:{k}_{L}a$) based on Higbie in LB-LES was 517 h^− 1^, which is higher than the RANS estimate of about 400 h^− 1^ and closer to the expected operational range of 600–750 h^− 1^ [8]. The increased *k*_*L*_*a* estimate using LB-LES may reflect the higher slip velocity of bubbles which improves *k*_*L*_ values.

Overall, E-E simulations remain the current state-of-the-art and are widely validated (e.g [10, 72, 73]), while E-L simulations have been applied for many years [74] for such simulations but are still less common. With the rise in artificial intelligence and the thereby increased availability of powerful graphical processing units (GPUs), the high parallelizability of operations in an E-L approach becomes increasingly powerful, making these simulations much more feasible nowadays while increasing the ability to consider turbulence more accurately. In order to make statements of the more suitable modelling approach in the biotechnology sector, experimental data would be needed which is still scarce for industrial-scale systems. Alternatively, direct numerical simulations might be performed. However, they are not suitable at this industrial scale to date. Both approaches reproduce comparable large-scale circulation patterns, but their local predictions of gas distribution, turbulence, and mass-transfer-relevant quantities remain model-dependent and require experimental validation. At this stage, the differences between the results of the two approaches are not attributed to single model features, such as underlying equations, the discretization schemes, turbulence modelling, or the bubble treatment. On contrary, all traits are interlinked creating a complex cause-effect relationship.

## Conclusion and outlook

In this study, we discussed different aspects of modeling pneumatically agitated bioreactors with CFD models. It was shown, that parcel sizes > 1 need to be considered for estimating hydrodynamics and mass transfer in industrial size bioreactors within reasonable computational time. Only in pilot scale, LB-LES can be performed with parcel size 1. However, applying the parcel approach comes along with estimating reduced mean liquid velocities and increasing *k*_*L*_*a* values. Interestingly enough, the coinciding use of the third law scaling exponent * <1* introduces the concept of swarm-like effects to the bubbles. The latter, however, is typically ignored in conventional E-E simulations although related impacts most likely exist. Our approach to estimate the impact of parcel sizes by making use of an exponential function with fixed impacts of parcel size but variable setting for the mean (reference) velocity may serve as a starting point for future LB-LES simulations.

In general, introducing the parcel concept for calculating large-scale bubble column hydrodynamics is crucial to perform manageable simulations, at least with current computational technologies.

## Supplementary Information

Below is the link to the electronic supplementary material.

Supplementary Material 1

## Data Availability

The simulation data is available in the repository https://doi.org/10.18419/DARUS-5525 (will be published upon acceptance).

## Abbreviations

BCR: Bubble column reactor
CFD: Computational fluid dynamics
CM: Compartment Model
EDR: Eddy dissipation rate
E-E: Euler-Euler approach
E-L: Euler–Lagrange approach
EL-ALR: External loop airlift reactor
FD: Finite difference
FE: Finite element
FV: Finite volume
LB(M): Lattice Boltzmann (Method)
LES: Large eddy simulation
PBM: Population balance model
RANS: Reynolds-averaged Navier–Stokes
STR: Stirred tank reactor

## List of symbols

*A*: Surface
${A}_{R}$: Cross-sectional area reactor
*a*: Specific interfacial surface
$${C}_{D}$$: Drag coefficient
$${C}_{S}$$: Smagorinsky coefficient
*d*: Number of spatial dimensions
$${D}_{L}$$: Diffusion coefficient
${d}_{p}$: Particle/Bubble diameter
*Eo*: Eötvös number
*f*: Density distribution function
$${f}^{eq}$$: Equilibrium distribution
$$\begin{gathered} F_{D} ,F_{L} , \hfill \\ F_{B} ,~F_{{VM}} \hfill \\ \end{gathered}$$: Drag/lift/buoyancy/virtual mass force on particle/bubble
${\overrightarrow{F}}_{f}$: Bubble forces acting on liquid
*g*: Gravitational acceleration
*k*: Kinetic energy
$${k}_{B}$$: Boltzmann constant
${k}_{L}$: Mass transfer constant
${k}_{L}a$: Mass transfer coefficient
${m}_{p}$: Mass of particle/bubble
*n*: Third law scaling exponent
$${N}_{p}$$: Parcel size
*p*: Pressure
*R*: Gas constant
*Re*: Reynolds number
*S*: Filtered strain rate tensor
*t*: Time
*T*: Temperature
$\dot{V}$: Liquid volume flow
$${V}_{0}$$: Stationary volume
$${V}_{L}$$: Liquid volume
${u}_{L},{\overline{u} }_{L}$: (Mean) Liquid velocity
${u}_{p}$: Particle/bubble velocity
${u}_{slip}$: Slip particle/bubble velocity
*x*: Position of particle distribution
$$\alpha , \beta$$: Fitting parameter
*Δ x*: Grid spacing
*\varepsilon*: Turbulent dissipation
$${\nu}_{e}$$: Eddy viscosity
*ξ*: Velocity of particle distribution
$${\rho}_{L}$$: Liquid density
*τ*: Stress tensor
$${\tau}_{rel}$$: Relaxation time
$$\Omega \left(f\right)$$: Collision operator
*ω*: Turbulent dissipation rate
$${\omega}_{L}$$: Fluid vorticity

## Footnotes

1
https://docs.mstarcfd.com/6_Create/Particles/txt-files/fluid-interaction.html#lift-saffman (accessed December 22, 2025).

2 M-Star Simulations, LLC, https://mstarcfd.com (accessed on December, 2025).

3 The MathWorks Inc. (2025). Curve Fitting Toolbox version: 15.1 (R2025a), Natick, Massachusetts: The MathWorks Inc. https://www.mathworks.com.
