# Supplementary material for: Simulating large-scale biotech bubble columns: challenges and insights from the parcel approach
Source: Bioprocess Biosyst Eng. 2026 Aug 8;49(9):2441–57. doi: 10.1007/s00449-026-03397-5 (PMC13582231; doi:10.1007/s00449-026-03397-5)
Supplement: Supplementary file 1 [file 449_2026_3397_MOESM1_ESM.docx]

**Supporting material**

**Simulating large-scale bubble columns: Curveballs and great opportunities**

Carolin Link^a^, Lars Puiman^b^, Ralf Takors^a,*^

*^a^University of Stuttgart, Institute of Biochemical Engineering, Allmandring 31, 70569 Stuttgart, Germany*

*^b^University of Queensland, Australian Institute for Bioengineering and Nanotechnology, Brisbane, QLD, Australia*

*^*^Corresponding author. E-mail address: takors@ibvt.uni-stuttgart.de (R. Takors)*

**Contents**

Supporting Material 1: Additional model information

Supporting Material 2: Independence studies

**Supporting Material 1: Additional model information**

Mid-way bounce back

In our model, the mid-/half-way bounce-back boundary condition was used. Bounce-back implies that the particle distribution reaching a wall scatters back to the original node where it came from (Chen & Doolen, 1998). The boundary is placed half-way in the middle of the last position and the wall node. A particle (bubble) is reflected at that boundary. For curved interfaces, the boundary is approximated as a step like boundary (Boyd et al., 2004). The parameters for the bounce are the following: Sliding Friction = 0.3, Rolling Friction = 0.1, Coefficient of Restitution = 0.5, Youngs Modulus (GPa) = 0.002, and Poisson Ratio = 0.3 (default in M-Star CFD and not editable in the commercial software). No particle-particle interaction is enabled since liquid-gas interactions dominate flow in bubble column reactors and contact forces in these applications are normally neglected (see e.g. (Mast et al., 2025; Muniz & Sommerfeld, 2020; Taborda & Sommerfeld, 2021). As in the Euler-Euler equivalent, collision forces of bubbles are neglected. The most important contact phenomena for bubbles are coalescence and breakup. However, as mentioned in the manuscript, these were neglected due to the small rigid bubbles and the increased ethanol concentration (Mast et al., 2025; Puiman et al., 2022).

Additional force

In Table 2 of the main article it was mentioned, that an additional force had to be added to prevent bubbles from being accumulated in certain parts of the reactor. This was necessary as the bubbles are considered as mass- and volume-less Lagrangian point particles. In horizontal parts of the tank, where the flow was insufficient to further push bubbles along and where buoyancy led to a rise of bubbles towards the wall, bubbles tend to accumulate. As they do not have a volume, critically high values of accumulated bubbles were reached in low velocity regions close to the walls, leading to bubble volume fractions above one. To prevent this unphysical behavior, a force was added. This force was only applied if the bubble volume fraction exceeded the physical limit of 1, and only in the critical regions with horizontal pipe parts ($y_{p}>18.6 \&\& y_{p}<22 \&\& x_{p}>3.4$ or $y_{p}<3.5 \&\& x_{p}>2.5)$. In those regions, bubbles were then accumulated in x- and negative y-direction to escape the endless accumulation ($F_{add,X}=15000\cdot u_{x,p}\cdot V_{p}\cdot\rho_{p}, F_{add,Y}=-15000\cdot u_{y,p}\cdot V_{p}\cdot\rho_{p}$). The margin of accumulation was found via trial-and-error. The force was then additionally restricted to absolute values between 1e-5 and 1 to prevent no movement at all in case the velocities are zero, or undefinable high forces.

Although this is arguably not a perfect solution to the problem of accumulating bubbles, it may be seen as bubbles being pushed away if the region is already accumulated with bubbles, so they do follow the flow and the physical forces again after this one rather unphysical push away from the critical region. Comparisons with modelling approaches where this was not applied (FV-RANS) showed similar flow results in those regions.

**Supporting Material 2: Independence studies**

Grid independence

The lattice independence studies evaluate the accuracy of the simulation results based on the grid resolution. However, considering Euler-Lagrange simulations, another restricting factor is the ratio between mesh cell size and bubble diameter which should be roughly around one to 10 (Evrard et al., 2021). This constraint is kept for LZ-values (number of grid points along the z-axis) up to around 235 in the present geometry. While values up to LZ 300 were tested, LZ 200 might be more suitable due to the additional constraint. All independence studies were conducted with a parcel size of 150.

Comparing results such as the vertical (y-)velocity and the predicted CO concentration across a pre-defined line in the tank, similar results were found for all resolutions in the riser (see Figure 1). While the velocities align well in the riser and are within the deviations over time, higher deviations were observed in the downcomer. For the CO concentrations, the values are overpredicted at the left wall of the tank, especially with the highest resolution. Apparently, bubbles got stuck in the small lattice closest to the wall in that case. The CO concentration predictions in the downcomer between resolutions of LZ 300 and 200 agree much better than those with LZ 100.

|  |  |
| --- | --- |
|  |  |
| Figure 1: Readout of time averaged vertical velocity (top) and CO concentration (bottom) on an output line in the riser (left) and downcomer (right) of an external loop airlift reactor CFD model at height 8 m. Different spatial resolutions with LZ 100 (dz = 0.07 m, **-**), LZ 200 (dz = 0.035 m, **-**), and LZ 300 (dz = 0.023 m, **-**) are compared. Values were averaged over 90 s after reaching a steady state. Mean values with standard deviations as error bars are given. | |

This investigation revealed that the intermediate spatial resolution of LZ 200 is most suitable due to the constraint mentioned by (Evrard et al., 2021), the ability to capture the CO concentration. Overall, deviations between the different resolutions is small.

Time step independence

Despite the independence of the spatial resolution, temporal resolution has to be investigated. The time step size should be chosen based on a compromise of higher accuracy (smaller steps) and lower computational costs (larger steps). The criterion of lattice densities with below 2% deviations (Mast & Takors, 2023) was fulfilled for all tested Courant numbers. Tested were Courant numbers of 0.001 (dt = 3.50e-5 s), 0.005 (dt = 1.75e-4 s), and 0.01 (dt = 3.50e-4 s).

While simulating 10 s needed around 12 h with the lowest Courant number, the intermediate time step size allowed this simulation in around 3 h, and for the lowest it took around 1.4 h.

The deviations in the readouts for the different time step sizes appear random due to the large turbulence in the system and not systematic. That being said, a Courant number of 0.005 was chosen, leading to results approximately matching those of the lowest Courant number while needing much less simulation time.

|  |  |
| --- | --- |
|  |  |
| *Figure 2: Readout of time averaged vertical velocity (top) and CO concentration (bottom) on an output line in the riser (left) and downcomer (right) of an external loop airlift reactor CFD model at height 8 m. Different temporal resolutions with Courant-numbers of 0.01 (dt = 3.50e-4 s,* ***-****), 0.005 (dt = 1.75e-4 s,* ***-****), and 0.001 (dt = 3.50e-5 s,* ***-****) are compared. Values were averaged over 90 s after reaching a steady state. Mean values with standard deviations as error bars are given.* | |

References

Boyd, J., Buick, J. M., Cosgrove, J. A., & Stansell, P. (2004). Application of the lattice Boltzmann method to arterial flow simulation: Investigation of boundary conditions for complex arterial geometries. *Australasian Physics & Engineering Sciences in Medicine*, *27*(4), 207–212. https://doi.org/10.1007/BF03178650

Chen, S., & Doolen, G. D. (1998). LATTICE BOLTZMANN METHOD FOR FLUID FLOWS. *Annual Review of Fluid Mechanics*, *30*(1), 329–364. https://doi.org/10.1146/annurev.fluid.30.1.329

Evrard, F., Denner, F., & van Wachem, B. (2021). Quantifying the errors of the particle-source-in-cell Euler-Lagrange method. *International Journal of Multiphase Flow*, *135*, 103535. https://doi.org/10.1016/j.ijmultiphaseflow.2020.103535

Mast, Y., Ghaderi, A., & Takors, R. (2025). Real Case Study of 600 m^3^ Bubble Column Fermentations: Spatially Resolved Simulations Unveil Optimization Potentials for l ‐Phenylalanine Production With *Escherichia coli*. *Biotechnology and Bioengineering*, *122*(2), 265–286. https://doi.org/10.1002/bit.28869

Mast, Y., & Takors, R. (2023). Transferring bubble breakage models tailored for Euler-Euler approaches to Euler-Lagrange simulations. *Processes*, *11*(4), 1018. https://doi.org/10.3390/pr11041018

Muniz, M., & Sommerfeld, M. (2020). On the force competition in bubble columns: A numerical study. *International Journal of Multiphase Flow*, *128*, 103256. https://doi.org/10.1016/j.ijmultiphaseflow.2020.103256

Puiman, L., Abrahamson, B., Lans, R. G. J. M. V. D., Haringa, C., Noorman, H. J., & Picioreanu, C. (2022). Alleviating mass transfer limitations in industrial external-loop syngas-to-ethanol fermentation. *Chemical Engineering Science*, *259*, 117770. https://doi.org/10.1016/j.ces.2022.117770

Taborda, M. A., & Sommerfeld, M. (2021). Modelling the Influence of Bubble Dynamics on Motion, Mass Transfer and Chemical Reaction in LES-Euler/Lagrange Computations. In M. Schlüter, D. Bothe, S. Herres-Pawlis, & U. Nieken (Hrsg.), *Reactive Bubbly Flows* (Bd. 128, S. 379–411). Springer International Publishing. https://doi.org/10.1007/978-3-030-72361-3_16
